# Urban Agriculture Interventions in Refugee and Immigrant Communities: A Scoping Review

**DOI:** 10.1007/s11524-025-00991-y

**Published:** 2025-08-04

**Authors:** Sophee Langerman, Nicolas Juarez, Ifrah Mahamud Magan, Odessa Gonzalez Benson

**Affiliations:** 1 University of Michigan, Ann Arbor, MI United States; 2 New York University, New York City, United States

**Keywords:** Urban agriculture, Community gardens, Refugees, Immigrants, Environmental interventions, Sustainability, Health outcomes

## Abstract

Urban agriculture, known as urban farming, urban gardening, or community gardening, has become an important avenue for community development, food security, and economic stability in response to increased urbanization. However, a less studied aspect of urban agriculture is its application for historically marginalized communities and refugee and immigrant communities specifically. Using a two-fold research question: What are the domains of application of urban agriculture interventions on refugee and/or migrant populations? What are the scales and geographic patterns of urban agriculture interventions? Following scoping review guidelines, 42 articles published from 1990 to 2024 were included after screening out 375 articles that were initially retrieved from the database search. Articles were examined based on the following criterion: population of interest, intervention type, intervention scale, and geography of author. Findings suggest five domains of application: well-being, physical health, ecological, economic, and sociological, the latter as the most common domain. Health, particularly mental health, was less evident in scholarship. In terms of scale and geography, findings suggest that studies about large-size interventions were mostly in the Global South (Middle East and African regions specifically), and studies on small and medium-sized interventions were in the Global North (United States, Canada and Australia specifically). For theory, findings point to two broad theoretical domains: relationality and materialist, and less attention to food and environmental justice. These findings raise questions pertaining to access to resources insofar as resources determine the scale/size of interventions and thus their application. Issues pertaining to health and food and environmental justice were applications that largely did not emerge in the data, raising questions for further research.

## Introduction

Urban agriculture, known as urban farming, urban gardening, or community gardening, has become an important avenue for community development, food security, and economic stability in response to increased urbanization (Reynolds & Cohen, 2016). Researchers recognize the role of urban agriculture as an important intervention for populations, and a tool for building community strength and connectivity in urban spaces (Reynolds & Cohen, 2016). Urban spaces are densely populated and have a high concentration of human infrastructure, such as buildings, parks, and public transit, in comparison to rural spaces. Research indicates that urban agriculture has many public health benefits in urban spaces, such as increased physical activity, increased consumption of fruits and vegetables, and lower rates of noncommunicable diseases (Mok et al., 2014). The scale of these interventions ranges from more local, small scales on specific households [19] to medium-scale community interventions [48] to larger, citywide interventions [2]. Specifically, communities in densely populated, marginalized, or disenfranchised urban neighborhoods benefit from having access to urban gardens and farms as a response to food insecurity by providing access to fresh fruits and vegetables for families with insufficient income (Orsini et al., 2013).

A less studied aspect of urban agriculture is its application for refugee and immigrant communities specifically. Urban agriculture interventions popularized in much of the Global North, especially the United States, have focused almost exclusively on food production [46, 61], without recognizing other aspects that may be relevant to refugees and immigrants. Even recent systematic reviews which have explicitly looked at benefits beyond food production do not investigate the utility of urban agriculture for refugee and immigrant communities [8, 47]. Meanwhile, studies have illustrated how these communities have unique features and needs in terms of food, food justice, and food security, and their relationship to land, language, space, time, and shared histories has also been examined within the context of urban farming intervention (Coté, 2016).

**Fig. 1:**
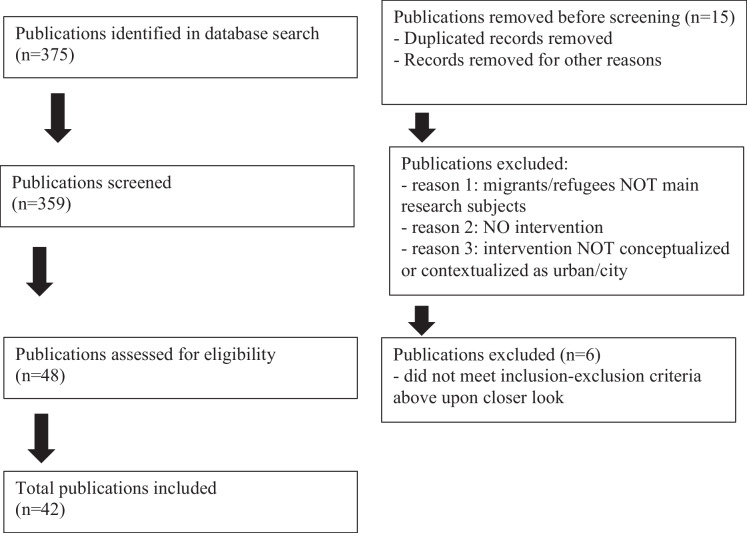
PRISMA flow diagram for scoping review

This scoping review focuses on urban agriculture interventions for refugee and immigrant communities. We apply a scalar and geographical analysis, examining the global scale (countries of interventions) and the local scale (national, regional, city, community/neighborhood). For methodology, we drew on the framework for scoping reviews as presented by Levac and colleagues (2010) (Fig. 1).

## Background

### Urban Agriculture

Urban agriculture is varied in terms of definition and in practice, particularly by region or location. In the context of North America, it is often viewed as plots of land within urban or peri-urban spaces dedicated towards growing fruits, vegetables, or cultivating livestock (Tornaghi, 2014). Urban agriculture includes aquaculture, agriculture, animal husbandry, and horticulture (Bowes et al., 2019). Operations vary in size and scale, which depend largely on community involvement and need, allotted space, and availability of funding.

The significance of urban agriculture took off in the 1980 s, with families and communities around the world reporting participation in some form of urban agriculture, though it has historically been used, even in ancient civilizations, as a method to keep communities fed (Smit et al., 1996). Backyard gardens and community gardens started reappearing around this time in conjunction with the rise of environmental movements, as people became more concerned with what they were eating and its impact on the environment (Mok et al., 2014). As urbanization increases, urban agriculture has been approached as an ideal intervention for food insecurity because of its community connection, sustainable foundation, and ability to adapt (Wurwarg, 2014). Countries experiencing hardships with food access have routinely relied on urban agriculture interventions to provide their communities with food and economic stability (Zezza and Tasciotti, 2010).

#### Urban Agriculture in Terms of Scale and the Global Context

The scale of urban agricultural interventions ranges from local household interventions to interventions that affect whole cities. Specific interventions at the scale of households can range from individual household gardens [40] to the conversion of home rooftops into garden spaces [3]. Alternatively, large-scale interventions exist, such as large-scale urban aquaponic programs [63] or city-wide food forests [42]. In between these two types of projects lie medium-sized interventions such as community gardens [30]. Differences in scale often relate to different funding sources and different actors, with smaller scales generally being done by individual citizens and larger-scale actions generally coming from governments or prominent non-profit organizations.

Globally, the political ecology of urban agriculture is often dependent on geographic location. Urban agriculture exists differently within communities in the Global North and Global South, where there is a boundary between a sort of leisure gardening experience and gardening out of necessity for food or income (Tornaghi, 2014). The relationship between geography, political dynamics, and the existence of urban agriculture is an important piece of contextual information. Urban agriculture is related to land ownership, the presence of money, time and capacity for a hobby, and something people engaged in alongside well-funded greening projects for affluent communities. In another regard, urban agriculture can be a space for contesting racial, class, and gender norms, rooted in radical organizing by people needing to grow their own food to survive [49, 51, 54].

#### Urban Agriculture in Marginalized Communities

Historically marginalized communities are greatly impacted by food policies that require an intervention [9, 13]. Food insecurity experienced in peri/urban areas is systemic and exacerbated by various factors, including extreme poverty and neighborhood segregation. In addition to environmental, health, and economic benefits, urban agriculture created space to challenge racial, class, and gender norms and promote environmentally just pursuits (Alkon & Agyeman, 2011,[52],Tornaghi, 2014). Innovative grassroot movements that address food deserts or food apartheids in predominantly Black and Brown neighborhoods do so by incorporating urban agriculture interventions and can be met with harsh repercussions as a result of restrictive city ordinances (Reynolds & Cohen, 2016). Moreover, implementing urban agriculture in marginalized communities in cities are often met with barriers such as access to land, infrastructure and resources in terms of time and money, as well as lack of agricultural knowledge and skills. Marginalized communities lack the resources, both human resources like time and capacity, and also material resources, like land.

Refugee and immigrant communities can find connectivity and resilience by embarking in projects and collective resistances that broaden individual experiences to shared histories [29, 33, 64]. Establishing a healing process rooted in familiarity associated to food and growing food to recover from the trauma experienced prior to and during resettlement, having a routine and agency over that routine or a physical space can help facilitate this healing. Autonomy and agency remain important tools in recovering from traumatic experiences, and collective projects have been identified as opportunities for immigrant and refugee community members to explore a new sense of agency or control [45].

### Theories on Urban Agriculture

Urban agriculture has been examined using theoretical perspectives related to its social and environmental dimensions, as well as via a justice lens. Researchers tend to use social theories to consider the ways in which urban agriculture can contribute to social cohesion [43] or to understand social inequality (Nazuri, 2022). Carvalho and Bógus [10], for example, utilize feminist critical theory to understand women’s participation in urban agriculture and to illuminate how gendered inequality contributes to food insecurity that urban agriculture might resolve. Social theories are therefore deployed by scholars of urban agriculture to understand the social dimensions of issues such as food insecurity or health disparities, as well as to understand how urban agriculture might assist in promoting social well-being beyond easily definable metrics such as economic income.

At the same time, urban agriculture researchers often attempt to understand the ways in which environmental or non-human factors affect projects beyond serving as either tools for human actors or as external constraints. Power (2005), for example, looks at how suburban gardeners’ interactions with specific trees and flowers in their gardens exemplified the plants’ agency. Rather than seeing plants as purely passive, gardeners often felt enticed or compelled by the behavior of the plants themselves. Other researchers [12, 31, 36] have similarly examined how the materiality of the environment possesses determinative capacity in urban agricultural projects.

Additionally, food justice and environmental justice perspectives have been used to examine urban agriculture. Specifically, research connects or theorizes urban agriculture in terms of its role in addressing issues of food deserts or food apartheids and environmental issues in predominantly Black and Brown neighborhoods [13, 37] Reynolds & Cohen, 2016,Tornaghi, 2014). These approaches emphasize the necessity of not leaving racial justice and systemic racism unthought in urban agricultural projects.

## Methods

### Research Questions

The research questions for this scoping review are two-fold: What are the domains of application of urban agriculture interventions on refugee and/or immigrant populations? What are the scale/size, geography, and informing theories of those urban agriculture interventions?

### Search: Identifying Relevant Studies

We searched for articles published in 34 years between 1990 and 2024 in the Scopus, Agricola, Families and Society, and Social Service Abstract databases. The initial search yielded 375 journal articles total. We used the following search terms: “Urban agriculture” or “Urban farming” or “Community gardens” or “Peri-urban agriculture” or “Urban gardening” or “urban gardens” or “Alternative farming” or “Community gardening” or “Alternative food systems” or “Rooftop gardening” or “rooftop gardens” or “Community-based agriculture” or “Vertical farming” or Sustainability or “sustainable agriculture” and “immigrant” or “refugee”.

### Study Selection: Inclusion/Exclusion Criteria

Articles were assessed for meeting inclusion criteria based on four things. First, articles were excluded if the population of interest were not refugees and/or immigrants as specified in the articles. Refugees were defined as individuals who were forced to flee their home country due to persecution and violence and have received official designation as refugee status by the United Nations High Commissioner for Refugees; immigrants were defined as individuals who moved either temporarily or permanently from another country due to reasons of economics/employment and/or family reunification. Refugees and immigrants tend to have a more secure residency status, compared with asylees, undocumented immigrants, and other forced migrants. Definitions for “immigrants” vary, and as a limitation of our study, we limited our review to immigrants and refugees, given different circumstances and privileges with respect to residency status. Second, articles were excluded if there was no specific intervention related to urban agriculture, as defined in the sections above. Articles were excluded if they referred to an urban agriculture intervention only briefly in the Introduction or Discussion sections and did not provide analysis of the intervention as the focus of the research. Third, articles were excluded if the intervention was not conducted in and designed for a specific urban area or city. Limiting to the urban/city is appropriate, given that interventions without specific mention of the city environment in which they were conducted in would have vastly different intent or aims, levels of resources, and mechanisms for implementation. Finally, articles excluded were those not written in English, not on urban agriculture at all, and not peer-reviewed (i.e., reports, gray literature). Initial determinations of exclusions were done by one researcher and then reviewed by a second researcher. Specifics for each inclusion/exclusion criterion were refined iteratively as the review progressed; that is, we conducted deliberations to re-define and re-conceptualize what would be included and excluded as informed by the articles reviewed and as the review process.

### Charting the Data

Then, for charting the data (Table 1), the 42 articles included in the study were analyzed in depth by two reviewers to identify application, scale/size, and geography of the intervention as specified or determined in the study, as well as specific aims, methods, data source, sample, findings, and theories. Contradicting or unparallel reviews were resolved through discussion. Based on the literature review, sensitizing concepts were used for coding application or outcomes of the articles, such as mental health, physical health, community, food security, economic stability, equity, consciousness raising, and racial justice. Coding also allowed for emergent codes, whereby applications or outcomes not based on existing literature could be added inductively or modified. As such, our coding process started with a deductive approach by using sensitizing concepts drawn from literature, but we later used a more inductive approach whereby we allowed for emergent codes. For our thematic analysis for “domains of impact” of interventions, we sought themes that would capture each article and thereby serve as a synthesizing framework to describe the whole set of articles included in the sample, while also considering parsimony or limiting the number of themes/domains so that they also serve to summarize the articles/data. For instance, while our initial code was mental health, we re-conceptualized this to be “overall well-being” as this broader concept better captured some of the explicit outcomes such as self-reliance. Further, the domains were not discrete concepts, so we coded articles for multiple domains. For instance, the sociological and economic domains often co-occurred.

**Table 1:** Findings

#	**Author**	**Study aims**	**Theory/framework**	**Target population**	**Specific application**	**Domain of application**
1	Abramovic et al. [1]	Discover how people engage in ecological experiences after resettlement	Material presence	Burmese refugees	Sense of belonging	Sociological
2	Bernholt et al. [5]	Assess plant diversity and peri-urban agriculture for genetic diversity conservation	None specified	Migrants (as well as Djerma ethnic groups)	Species composition, richness and diversity, family nutrition, income	Ecological, physical health
3	Bessho et al. [6]	Examine migrant involvement in community farms	Social inclusion of immigrants	Migrants	Willingness to support community members	Sociological
4	Blumberg et al. [7]	Reflect upon challenges and possibilities of community gardening in peripheral cities	Feminist geography, food justice	Latino migrants	Physical health, safety	Physical health, sociological
5	Buchthal et al. (2019)	Assess community gardening practices	None specified	Immigrants (Asia Pacific)	Changes and differences in land use practices across incomes	Ecological
6	Castro et al. [11]	Evaluate a community intervention in preventing childhood obesity	None specified	Latino migrants	Greater consumption of fruits and vegetables/healthy diet	Physical health
7	Clarke and Jenerrete (2014)	Investigate drivers of plant richness and abundance	None specified	Latino migrants, Korean migrants	Greater biodiversity	Ecological
8	Coughlan and Hermes [14]	Investigate role of green space in the experience of displacement and resettlement	None specified	Somali Bantu refugees	Mental health, community building, reconciliation	Well-being, sociological
9	Datta [15]	Explore the role of cross-cultural activities in community garden	Relationality	Refugees, migrants (and indigenous groups)	Communication, belonging, reconciliation skills, decolonization	Sociological
10	Dehnavi and Süß [16]	Highlight the impact of urban agriculture on food security	None specified	Syrian refugees	Economic resilience, food security	Economic
11	Dyg et al. [17]	Document effects of community gardens on well-being	None specified	Refugees, migrants	Physical activity, diet, food knowledge, independence, self-worth	Physical health, sociological
12	Eggert et al. [18]	Use Community Coalition Action Theory to implement refugee community gardens	Community Coalition Action Theory	Refugees	Financial health, physical health, sense of agency and mental health	Economic, well-being, physical health
13	Gerber et al. [21]	Document effects of community gardens on vulnerable populations	None specified	Refugees	Mental health, self-efficacy, autonomy, social support	Well-being, sociological
14	Ginn and Ascensao (2018)	Explore subaltern urbanism and top-down commons in an urban gardening setting	None specified	Black migrants from former colonies	Autonomous, bottom-up commons	Sociological
15	Girbés-Peco et al. [22]	Evaluation of a community garden project in reversing inequalities	Participation, social capital, power, relationships	Morroccan migrants	Job skills, social networks and knowledge sharing	Economic, sociological
16	Gray et al. [23]	Examine home-garden project in immigrant neighborhoods	None specified	Latino migrants	Finances, consumption, physical activity, connectedness	Economic, physical health, sociological
17	Harper (2015)	Understand resilience in emancipatory framework in community gardens	Social–ecological resilience	Karen refugees	Resilience, self-sustaining knowledge, comfort, education	Sociological
18	Harris et al. [24]	Explore how community food gardens support connections and humanitarianism	None specified	African refugees	Sense of belonging, connectedness to country	Sociological
19	Hartwig and Mason [25]	Evaluate church-based community garden	None specified	Refugees, migrants (Karen, Hmong Bhutanese Lisu)	Food security, mental and physical health, social support	Economic, sociological
20	Head et al. [27]	Understand migrant engagements with sense of place	None specified	Macedonian, Vietnamese migrants	Increased vegetable growth	Ecological, economic, sociological
21	Head et al. [26]	Examine environmental engagement through ethnic minority migration lens	None specified	Migrants	Mental wellness, productivity, connection to nature, memory	Well-being, sociological
22	Heraty and Ellstrand [28]	Investigate plant richness, abundance and production in community gardens	None specified	Hispanic migrants, Korean migrants	Maize crop biodiversity	Ecological
23	Hondagneu-Sotelo (2015)	Explore recreation of homeland through urban community gardens	Persistent materiality	Latino migrants	Feelings of home, social reproductive activities	Sociological
24	Knigge [32]	Investigate social services provided by community gardens	None specified	Refugees	Care and support, role of african american women	Sociological
25	Lintner and Elsen [35]	Investigate social cooperatives and agricultural social work	None specified	Refugees, aslyum seekers	Co-production of crops, sustainability, community empowerment	Ecological, sociological
26	Millican et al. (2018)	Understand links between gardening/green space and mental health and trauma	None specified	Syrian refugees	Environmental benefits, mental/physical health, sense of belonging	Ecological, sociological
27	Minkoff-Zern [38]	Examine farmworkers’ coping with food insecurity through agricultural knowledge	None specified	Latino migrants, indigenous people (Oaxaca, Mexico)	Food security, reclaiming cultural knowledge and practices, pride	Economic, sociological
28	Moore et al. [39]	Identify agroforestry preferences	None specified	Refugees, Central African Republic refugees (CAR)	Response to environmental challenges	Ecological
29	Nail [41]	Explore urban greening as a process of memoralisation and social cohesion	None specified	Refugees, displaced people (Colombia)	Food security, well-being, traditional knowledge, social networks	Economic, well-being, sociological
30	Piekielek [44]	Explore sustainable development through cooperativism	None specified	Japanese migrants	Ecological knowledge, ethnic identity, cooperativism	Sociological
31	Saldivar-Tanaka and Krasny (2003)	Determine how community gardens relate to community development, civic agriculture	None specified	Latino migrants	Social interactions, landscape design, organizing experience, community development	Sociological
32	Sandoval and Rodine [53]	Investigate links between integration and agricultural, environmental sustainability	None specified	Latino migrants	Food security, immigrant integration, community, sense of belonging	Economic, sociological
33	Shan and Walter [56]	Examine learning practices of gardeners across ethnic community gardens	Sociocultural and sociomaterial as lens for learning	Chinese migrants	Multiculturalism	Sociological
34	Tomkins et al. [59]	Examine homegarden emergence in refugee camps	None specified	Refugees	Autonomy, urbanism, remembrance	Sociological
35	Vandebroek and Balick [60]	Examine traditional medicine in disease prevention via community gardens	None specified	Dominican migrants (in food deserts)	Prevention of non-communicable diseases	Physical health
36	Weltin and Lavin [62]	Monitor diabetes among community gardeners	Care seeking behavior	Marshallese migrants	Cardiovascular health, exercise level, healthy diet, lower blood sugar	Physical health
37	Rogers et al. [50]	Evaluate urban agriculture youth development program	None specified	Immigrants	Leadership skills, agricultural skills, food insecurity	Overall well-being, economic
38	Castro et al. [11]	Evaluate gardening intervention for childhood obesity prevention	None specified	Latino Immigrants	Prevention of obesity	Physical health
39	Storm et al. [57]	Improve refugee collaboration and empowerment via gardening	None specified	Syrian refugees	Social cohesion, development of social capital, empowerment	Overall well-being
40	Gangamma et al. [20]	Examine gardening’s influence on refugee well-being	None specified	Refugees	Mental health, food insecurity	Overall well-being
41	Loos et al. (2023)	Analyze rooftop urban agriculture’s livelihood effects for refugees	None specified	Female, Palestinian refugees	Social capital, sustainability, food insecurity	Overall well-being, economic
42	Liddicoat et al. [34]	Explore the benefits of garden mosaics on immigrant youth	None specified	Immigrant youth	Cultural memory, social cohesion, ecological knowledge	Sociological, ecological

## Results: Collating and Summarizing Data

To collate and summarize data, Table 1 presents details about the articles, including a summary of study aims, population of interest, domains of applications, and theories, and we discuss thematic results below. The articles used varied terms: urban and peri-urban agriculture, community garden, backyard garden, urban greening, home garden, social and agriculture cooperatives, ranchitos, agroforestry, and micro-gardening.

### Five Domains of Application

Findings suggest five broad domains for the application of urban arboriculture interventions. Most articles report multiple domains; the number of articles for each domain is thus greater than the 42 articles in our sample.

### Overall well-being Applications (n = 11)

For the first subdomain, 11 articles specified various aspects of individual well-being, such as mental health [14, 25, 58],Millican et al. 2018), self-efficacy, and autonomy [21],Hondagneu-Sotelo, 2015). For instance, Jean (2015), Rogers et al. (2019), and Storm et al. [57] all illustrated how urban gardening was emotionally beneficial for refugee participants as it allowed them connections with others which then alleviated feelings of isolation in their new community. Gangamma et al. [20] suggests that these benefits may derive from the stronger social connections refugees develop through gardening and the opportunities that gardening provides to stay connected to one’s culture in a new place.

### Physical Health Applications (n = 11)

Eleven articles highlighted the physical applications of urban gardening on participants, such as the prevention of noncommunicable diseases (Vanderboerk and Balick, 2014), improved diet and nutrition, and increased physical activity [11, 17, 62], and improved nutritional health Eggert et al. [18], attributed to better access to more fruits and vegetables that would otherwise be too expensive Eggert et al. [18].

### Economic Applications (n = 13)

Third, individual economic applications in the 13 articles include food security [20, 38, 41], stable family income (Berholt et al., 2009,Rogers et al., 2019; [23]), self-sufficiency [27], and gaining skills for future employment [22].

### Sociological Applications (n = 29)

Examples of sociological applications include traditional knowledge sharing, reconciliation, memory and empowerment (Head et al., 2019; [34, 41, 57]). This domain is especially important given the historical and generational traumas that refugee and immigrant communities have been subjected to. In Tomkins et al. [59], Syrian refugees in the Kurdistan region of Iraq found remembrance of their home in creating home gardens. Similarly, immigrants in Sydney, Australia, maintained a connection to cultural traditions and histories through their backyard gardens [27].

### Ecological Applications (n = 8)

The fifth domain, in 8 articles, encompasses ecological applications, including an increase biodiversity and species richness of non-ornamental plants, coverage of edible plants (Clarke & Jenerrette, 2014), start up organic food production [35], different land use practices (Buchthal et al., 2019), and greater plant species diversity, richness, and composition [5]. In one study, Heraty and Ellstrand [28] underscore the value that biodiverse community gardens bring to the urban landscape of LA County, CA, where immigrant gardens had an overall greater biodiversity in maize crops than commercial production, and relied on traditional farming methods to sustain genetically diverse crops.

## Scalar and Geographic Patterns in Urban Agriculture Interventions

### Scale or Size of Interventions

Findings illustrate three sizes or scales of intervention: small-household, medium-networked and large-institutionalized, whereby the factors for determining scale were physical size and actors’ involvement or participation. Some articles tackled small-scale interventions, focusing on home gardens and micro-gardening interventions, typically serving one or two households, and being managed by a single family. Meanwhile, the majority of articles focused on medium-sized interventions, including community gardens, peri-urban agriculture, backyard gardens, and home gardens. These medium-sized interventions were often run by NGOs or local organizations, included gardening workshops and food purchasing programs, community gardens, small urban and peri-urban agricultural projects for a block or neighborhood. Finally, a few articles focused on large-sized interventions, typically taking place in entire communities or refugee settlements, with direct government involvement. Large-scale interventions included larger greening projects for public parks and spaces, in addition to urban agriculture for food access.

### Geography of Interventions

Global North countries such as the United States, Canada, and Australia were the most common study sites for small-sized interventions and medium-sized interventions, stemming largely from local initiative of the community or individual families. Meanwhile, the MENA region and West Africa region were the study sites for the majority of large-scale, institutionalized interventions, hosted by governmental agencies or in partnership with larger institutions and typically located in refugee settlements in areas within close proximity to displacement and forced migration. Examples include the Kurdistan-Region of Iraq, Lebanon, Niger, and the Republic of Cameroon.

### Theories Perspectives

Only 10 of the 42 articles offered a deepened discussion of theoretical perspectives and utilized two main frames based on the (a) social dimension and (b) environmental or non-human dimensions, supplemented by other theories considered as exceptions.

#### Theoretical Perspectives based on Social Dimensions

Theories pertaining to the social or relational dimensions of urban gardens were common, utilized in five of the 10 articles. First, immigrant social inclusion was a conceptual tool towards a more holistic perspective that allows us to think about urban gardens as a part of inclusion efforts (Bessho and colleagues (2018). Second, the Community Coalition Action Theory was used in examining community coalitions in the context of urban gardens (Butterfoss and Kegler, 2009; [18]). Third, participation, social capital, and power were underpinning concepts for examining a school’s community gardening [22]. Fourth, sociocultural theories of learning intertwined with sociomaterial theory showed how social activities of learning and practicing are mediated through “cultural artifacts or cultural tools such as language, texts, and technology” [56]. Fifth, using the concept of relationality, cross-cultural activities in community gardens were examined as mechanisms through which the connections between the material and spiritual are forged [15].

#### Theoretical Perspectives based on Environmental or Non-Human Dimensions

Theories on the presence/importance of the non-human, environment or.

material dimensions were used in five articles. As a set, these articles view relationships with community gardens as extending beyond the human to include ecological factors, particularly gardeners’ engagement of land, soil, climate, and weather alongside people. These articles used various concepts and conceptual claims: material presence [1], social-ecological resilience (Harper, 2015), “persistent materiality” and “home as a site of belonging beyond the individual household” (Hondagneu-Sotelo, 2015), “see(ing)cognition and agency also (in) nonhuman” [56], p. 22), and a focus “not only actors but actions as well” within the context of nature and land [15], p. 763).

#### Other Theories: Critical Theory, Behavioral Theory, Pedagogical Theory

First, a *critical perspective, related to feminist geography, food justice*, *and power*, was used in two exception articles. Blumberg and colleagues (2019) analyzed food justice organizing nodes as a way for activists and scholars alike to create pathways leading to transformative change. Girbés-Peco and colleagues (2020) meanwhile drew upon power analyses to inform the evaluation of a community garden project in reversing inequalities. Second, the *theory of care-seeking behavior* was used in one exception article to argue for participating in a community garden as “healthy behavior” that “is a function of psychosocial variables (affect, expectations, and values about outcomes, habit, and norm)” [62], p. 14). Third, Datta (2015) drew on *pedagogic or learning theory* to examine community gardens as a site of learning about ecological aspects, but also about things such as the politics of space, decolonization of place, and participatory democracy (Datta, 2015).

## Discussion

This scoping review illustrates the varied applications of urban agriculture specifically for refugee and immigrant communities: overall well-being, physical health, economic.

sociological, and ecological. Importantly, we want to clarify that our review did not assess for impact or effectiveness of interventions and instead examined only what domains the interventions *explicitly sought to* impact, which we termed as domains of application.

Notably, sociological or community-level was the most common domain of impact, with more than double the number of articles compared with other domains. Relatedly, medium-sized interventions at the community level (versus national level and family level) were the most common scale or size of interventions. Research has illustrated that community solidarity and cooperation, as community-level applications, are salient for refugees and immigrants, particularly during early phases of arrival and integration [4],Martén, Hainmueller & Hangartner, 2019; [55]. Our findings lend support for these perspectives and expand the literature by adding urban agriculture as a specific intervention for these community-level applications.

However, illustrating the flip side of the point above, our findings also suggest there is less academic attention to health, as well as the economic and ecological, as the other domains impacted by urban agriculture. Notably, mental health was largely missing from the findings, compared with overall health and physical health. These findings are somewhat surprising, given that health, even as distal outcome, seems fundamentally linked with ecology and food security as central to urban agriculture.

Particularly at the scale of the urban, health is deeply intertwined with the structural aspects that co-constituted the city, such as access to food, income security, and ecological adaptation, that could in turn be linked to urban agriculture interventions. Urban agriculture requires land as a physical resource, thus overlapping with housing, which is often a central urban problematic. Further, urban agriculture mandates human resources, necessitating time, skills, and capacity from those involved. Income security, labor, and economic supports as urban issues thus come into play. Housing and income security, in turn, impact health. And, of course, local ecology—at the heart of urban agriculture—is also an integral aspect of urban planning. In the context of newcomers, it is thus vital to also examine and understand urban agriculture through a holistic lens that factors in not only access to food and urban agricultural interventions but also access to quality housing, education, income security, and healthcare for newcomer populations. These links to broader urban systems may help identify how, if at all, urban agriculture may improve outcomes for immigrant and refugee populations.

Further, findings were insightful with regard to the geographic aspect to urban agriculture interventions for refugee and immigrant communities. Large-size interventions were mostly in the Global South (MENA and West Africa specifically), while small and medium-sized interventions were in the Global North (United States, Canada and Australia specifically). Large-size interventions were funded by international NGOs or government agencies, while small and medium-sized interventions emerged from local efforts and local resources. Thus, these findings raise questions pertaining to access to resources insofar as resources determine the scale/size of interventions and thus their applications. Taking account of these findings that pertain to the global scale, urban health research may perhaps turn to the Global South for knowledges and approaches that can be learned and gained in terms of large-size interventions.

Interestingly, food justice and inequality were theoretical frames that did not emerge as dominant in the data, evident in only two articles that we thus consider as exceptions. Food and environmental justice have been used in studies of urban agriculture writ large as discussed in the background section above. However, our findings did not lend support for their application to studies on urban agriculture with refugee and immigrant communities specifically. This is surprising, given that other studies have illustrated how urban agriculture can contribute to greening and esthetics of a place, support local production and engagement of communities in the production of their own food, and reduce the emissions of greenhouse gases that occur with shipment of foods. This thus presents as missing theoretical or discursive framing, whereby academic attention has neglected to address issues of justice, specifically in the context of urban agriculture with refugee and immigrant communities. Further studies could look more in depth at the theories and characteristics of the interventions themselves, specifically in terms of the level of participation of refugees and immigrants themselves. It is one thing for interventions to be conducted *on* refugees and immigrants, versus *with* refugees and immigrants. Further studies could examine power dynamics and level/quality of participation or inclusion of refugees and immigrants in the design and implementation of urban agriculture interventions.

## Conclusion: Implications and Future Directions in Urban Health

Our review of the literature points to the vital role urban agriculture plays in fostering a sense of community in immigrant and refugee groups. For newcomers, access to quality food is essential, but urban agricultural spaces also afford a sense of connection, belonging, and solidarity among groups. These contributions are essential in the post-resettlement integration of immigrant and refugee communities. Given that health (as well as economic and ecological) domains did not feature prominently in our findings, future research should examine the ways in which urban agriculture is connected to broader urban systems, including healthcare, and how urban agricultural spaces may contribute to health outcomes of refugee and immigrant populations. In particular, studies should examine the role of urban agriculture as it relates to mental health outcomes, as an area that was not covered in the set of articles reviewed. Given that food and environmental justice as a theoretical lens did not feature prominently in our findings, future research may also examine the role of structural and systematic racism in the availability of, and access to, urban agricultural spaces for immigrant and refugee communities, in connection with broader histories of land and food justice among Indigenous, Black, and Brown communities in the United States.
